# Characterization of EBT3 radiochromic films for dosimetry of proton beams in the presence of magnetic fields

**DOI:** 10.1002/mp.13567

**Published:** 2019-05-31

**Authors:** Fatima Padilla‐Cabal, Peter Kuess, Dietmar Georg, Hugo Palmans, Lukas Fetty, Hermann Fuchs

**Affiliations:** ^1^ Department of Radiotherapy Medical University of Vienna/AKH Vienna Austria; ^2^ Christian Doppler Laboratory for Medical Radiation Research for Radiation Oncology Medical University of Vienna Vienna Austria; ^3^ EBG MedAustron GmbH Wiener Neustadt Austria; ^4^ National Physical Laboratory Teddington TW 11 0LW UK

**Keywords:** EBT3 films dosimetry, magnetic fields, proton therapy

## Abstract

**Purpose:**

Radiochromic film dosimetry is extensively used for quality assurance in photon and proton beam therapy. So far, GafchromicTM EBT3 film appears as a strong candidate to be used in future magnetic resonance (MR) based therapy systems. The response of Gafchromic EBT3 films in the presence of magnetic fields has already been addressed for different MR‐linacs systems. However, a detailed evaluation of the influence of external magnetic fields on the film response and calibration curves for proton therapy has not yet been reported. This study aims to determine the dose responses of EBT3 films for clinical proton beams exposed to magnetic field strengths up to 1 T in order to investigate the feasibility of EBT3 film as an accurate dosimetric tool for a future MR particle therapy system (MRPT).

**Methods:**

The dosimetric characteristics of EBT3 films were studied for a proton beam passing through magnetic field strengths of B = 0, 0.5, and 1 T. Absorbed dose calibration and measurements were performed using clinical proton beams in the nominal energy range of 62.4–252.6 MeV. Irradiations were done using an in‐house developed PMMA slab phantom placed in the center of a dipole research magnet. Monte Carlo (MC) simulations using the GATE/Geant4 toolkit were performed to predict the effect of magnetic fields on the energy deposited by proton beams in the phantom. Planned and measured doses from 3D box cube irradiations were compared to assess the accuracy of the dosimetric method using EBT3 films with/without the external magnetic field.

**Results:**

Neither for the mean pixel value nor for the net optical density, any significant deviations were observed due to the presence of an external magnetic field (B ≤ 1T) for doses up to 10 Gy. Dose‐response curves for the red channel were fitted by a three‐parameter function for the field‐free case and for B = 1T, showing for both cases an R‐square coefficient of unity and almost identical fitting parameters. Independently of the magnetic field, EBT3 films showed an under‐response as high as 8% in the Bragg peak region, similarly to previously reported effects for particle therapy. No noticeable influence of the magnetic field strength was observed on the quenching effect of the EBT3 films.

**Conclusions:**

For the first time detailed absorbed dose calibrations of EBT3 films for proton beams in magnetic field regions were performed. Results showed that EBT3 films represent an attractive solution for the dosimetry of a future MRPT system. As film response functions for protons are not affected by the magnetic field strenght, they can be used for further investigations to evaluate the dosimetric effects induced due to particle beams bending in magnetic fields regions.

## 1 Introduction

Radiochromic film dosimetry has been used extensively in the last years for dose verification and to measure 2D dose distributions in brachytherapy, photon and particle beam therapy.^1^, ^2^, ^3^, ^4^ Gafchromic^TM^ EBT films, specifically designed for external beam therapy, consist of an active monolayer (28 μm thickness) coated from both sides with two matte‐polyester layers of 125 μm thickness, resulting in an average mass density of 1.2 g cm^−3^ and a thickness of less than 0.3 mm.^1^, ^5^, ^6^ The high spatial resolution, small energy dependence, and the negligible perturbation of the radiation field at measurement positions within calibration phantoms are some of the main advantages of Gafchromic^TM^ EBT films for accurate absorbed dose determination.^3^, ^7^

Recently developed Magnetic Resonance Image (MRI)‐guided treatment machines using either Co‐60 sources or 6 MV linear accelerators have challenged conventional dosimetry when typical irradiation fields interact with the external magnetic fields from the MRI core. Several studies demonstrated that magnetic fields influence the response of standard detectors used for absolute dose verification in photon therapy.^8^, ^9^, ^10^, ^11^ Gafchromic^TM^ film dosimetry in MRI‐guided x‐ray beam therapy (MRXT) was also studied recently.^12^, ^13^, ^14^ Small but significant modifications in the net optical density and dose‐response curves were found for EBT3 and EBT2 films exposed to different dose levels of photon irradiations in magnetic field with strengths of up to 1.5T.^12^, ^13^, ^14^

After the promising results and clinical implementation of real‐time MRXT, the idea of an integrated system using proton beams (MRPT) has stimulated dedicated research in the last years.^15^, ^16^, ^17^ In particle therapy previous works^18^, ^19^, ^20^ showed that, although magnetic fields induce dose distortions due to the beam lateral deflection, MRPT is feasible from a dosimetric point of view with the implementation of proper beam arrangement corrections. These preliminary studies focused on the influence of the magnetic field on dose delivery and calculation; however , not on its influence on dose verification measurements.

Although Gafchromic^TM^ EBT film dosimetry is widely used in particle therapy for absolute dose and verification measurements,^21^, ^22^, ^23^ the suitability and accuracy of this method in the presence of external magnetic fields has not yet been investigated. This work aims to study the feasibility of EBT3 film dosimetry for a possible future MRPT system. For this purpose, the influence of external magnetic fields on the dose response of the detectors in proton therapy was carefully analyzed and compared with previous results reported for MRXT.

## 2 Materials and methods

### 2.A Experimental setup

**Figure 1:**
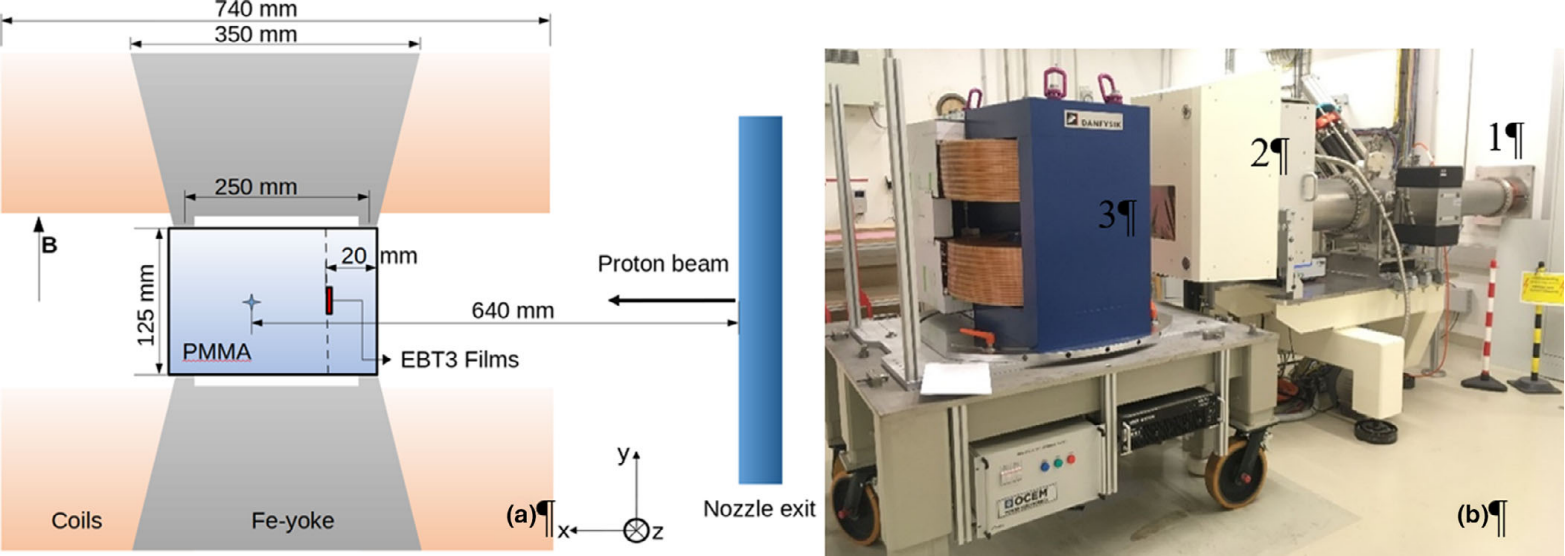
Sketch of the experimental setup (a) used for the irradiation of films within a magnetic field region, where the room isocenter position is represented by the blue star. Picture of the experimental setup at MedAustron (b) showing the beam line (1), the retractable Nozzle (2), and the dipole magnet (3) placed at the room isocenter. [Color figure can be viewed at http://wileyonlinelibrary.com]

Measurements were performed using a horizontal pencil beam scanning proton beam line in the dedicated research room at the MedAustron ion beam therapy center (EBG MedAustron GmbH, Wiener Neustadt, Austria).^24^ A dipole research magnet (Danfysik A/S, Taastrup, Denmark) was positioned in the isocenter of the research room, as shown in Fig1, generating a static, homogeneous magnetic field perpendicular to the beam incidence direction. Magnetic fields ranging from 0 to 1 T can be obtained by changing the nominal current. A high‐homogeneity magnetic field region (deviations from nominal value <0.1%) is achieved in a pole gap of 125 mm and a radial distance of 75 mm, according to measured data supplied by the manufacturer. An AS‐NTM Transverse Probe coupled to a FM 302 Teslameter (Projekt Elektronik Mess‐ und Regelungstechnik GmbH, Germany) was used to measure magnetic field intensities inside and outside the research magnet. An in‐house built solid PMMA slab phantom with dimensions 200 mm^3^ × 120 mm^3^ × 300 mm^3 ^was carefully placed in the center of the magnet, assuring that during irradiations the externally applied field was homogeneous within the entire volume. Proton beam energies in the range between 62.4 and 198 MeV corresponding to water ranges from 30 to 250 mm were used. EBT3 films were always placed in the center of the slabs transverse to the beam incidence. Large field sizes of 100 mm^2^ × 80 mm^2^ were used to guarantee homogenous irradiations of the films while avoiding the irradiation of the magnet poles. In order to assess the effect of external fields on proton dose distributions, identical irradiations were performed with and without magnetic field.

### 2.B Film irradiations and analysis

Gafchromic^TM^ EBT3 films sheets (lot # 06291702), cut into 40 mm^2^ × 30 mm^2^ pieces, were used throughout all measurements. Film handling methods were performed according to previous established experience in our research group.^2^ The films were positioned in the center of the proton beam in a stack of three pieces each and the results were hereafter averaged. The same orientation of the films within the magnetic field was kept during all the irradiations, with the short film side parallel to the magnetic field.

#### 2.B.1 Absorbed dose calibration

For their calibration in terms of absorbed dose to water, films were placed in the PMMA phantom in the plateau region at a reference depth of 20 mm in PMMA (corresponding to a water‐equivalent depth of 24 mm) and exposed to seven different dose levels (0, 0.2, 0.5, 1, 2, 5, 10 Gy) using a 148.2 MeV proton beam and a field size of 100 mm^2^ × 80 mm^2^. Although the IAEA TRS 398 guidelines^25^ as adapted to scanned beams^26^ were followed, measurements were performed at 20 mm in a solid PMMA phantom instead of water. The lack of a water phantom fitting between the magnet yokes and the impracticality of chamber positioning in this restricted volume did not allow performing the calibration procedures in water. Nevertheless, all dosimetric measurements were performed using the same phantom configuration assuring a constant water to PMMA fluence correction factor.^27^ The absorbed dose values at the above‐mentioned reference depth were also determined with the magnet switched off using a calibrated Roos chamber (PTW type 34001) connected to a UNIDOS electrometer (PTW, Freiburg, Germany). Magnetic fields strengths of 0, 0.5, and 1.0 T were used during the irradiations.

#### 2.B.2 Dose verification

The accuracy of the absorbed dose to water obtained from the EBT3 films was evaluated using two spread‐out Bragg peaks (SOBP). Irradiation plans were created using the treatment planning system (TPS) RayStation v5.99 (RaySearch Laboratories, Stockholm, Sweden) and recalculated with Monte Carlo simulations using the GATE/Geant4 toolkit. Two different box targets 100 mm^3^ × 80 mm^3^ × 40 mm^3^ centered at penetration depths of 75 and 125 mm from the phantom surface were irradiated in a single fraction with a prescribed dose level of 2Gy and magnetic fields of 0 and 1 T. Measurements were performed placing two film pieces of 40 mm^2^ × 30 mm^2^ in the center of the field, transverse to the beam incidence at ten different penetration depths and the Roos chamber at three different reference depths, covering the plateau and the Bragg peak region. The response of the Roos chamber at five different measurement depths (20, 60, 80, 110, and 130 mm) was also compared for both magnetic field strengths using the above‐mentioned 3D irradiation plans.

Considering the lack of a TPS accounting for magnetic field effects on particle dose distributions, Monte Carlo (MC) simulations were used to predict dosimetric differences between the irradiations at different magnetic field strengths (B = 0, 0.5, and 1 T). All the treatment plans used either for absolute dose calibration or for dose verification were recalculated using a developer version of GATE8.0/GEANT4.10.03.p01 toolkit including a proton beam line model and the three‐dimensional field map of the dipole magnet. The exact geometry of the experimental setup was reproduced in the MC model to determine the energy deposited by the beam alongside its path in the PMMA phantom. The number of particles per simulation was set to 10^7^ to ensure a type‐A uncertainty below 1% on our calculated absorbed dose values. All simulations were conducted on the in‐house computing cluster from the MOCCAMED group. Further details of the MC models can be obtained from previous works of our research group.^18^, ^19^

For dose verification, measurements were compared with the TPS calculated doses for the nonfield case and with Monte Carlo plan recalculations for the two extreme magnetic fields strengths of 0 and 1T.

#### 2.B.3 Film postprocessing

According to recommendations on EBT3 film evaluation including scanning protocols^4^, ^28^ the film analysis was performed 36 h after irradiation. The film pieces were scanned in landscape and portrait orientation in the center of an Epson 11000 XL flat‐bed scanner (Seiko Epson Corporation, Nagano, Japan). All scans were acquired in transmission mode, with 150 dpi (0.169 mm/pixel) resolution and saved as 48‐bits RGB color channel tagged images file format (tif). All available color correction options from the scanner were turned off.

Evaluations were done using an in‐house developed script in the commercial software MATLAB R2016b, (The MathWorks, Inc., Natick, Massachusetts, United States) for each of the individual color channels: red, green, and blue. For all the channels, mean pixel values (PV) and standard deviations were obtained in a square region of interest (ROI) of 20 mm^2^ × 20 mm^2^ around the centered beam axis and converted to net optical densities:.^3^

(1) $$OD_{\mathrm{net}}=\log\frac{PV_{\mathrm{bkg}}+1}{PV_{\mathrm{irr}}+1}$$

where the PV were calculated for the scanned images before ($PV_{\mathrm{bkg}}$) and after ($PV_{\mathrm{irr}}$) the irradiations.

For each measurement point, final results were obtained averaging the corresponding values of the three films used for each irradiation. Additionally, the signal‐to‐noise ratio (SNR) was evaluated by the ratio of the mean PV and the standard deviation for different dose levels and magnetic field strengths, respectively. Experimental uncertainties for the net optical densities were obtained by propagation of the uncertainties on the mean pixel values and the standard deviations over the three films.

Dose‐response curves were obtained for the two most extreme magnetic field intensities, 0 and 1T following the criteria suggested by Devic et al.^4^ and fitted by a three‐parameter function:

(2) $$y=ax+bx^{c}$$

Dose estimations were compared for all magnetic field intensities and the difference between measured and fitted values used as assessment parameter of the method accuracy.

## 3 Results

### 3.A Magnetic field influence on depth dose distributions

**Figure 2:**
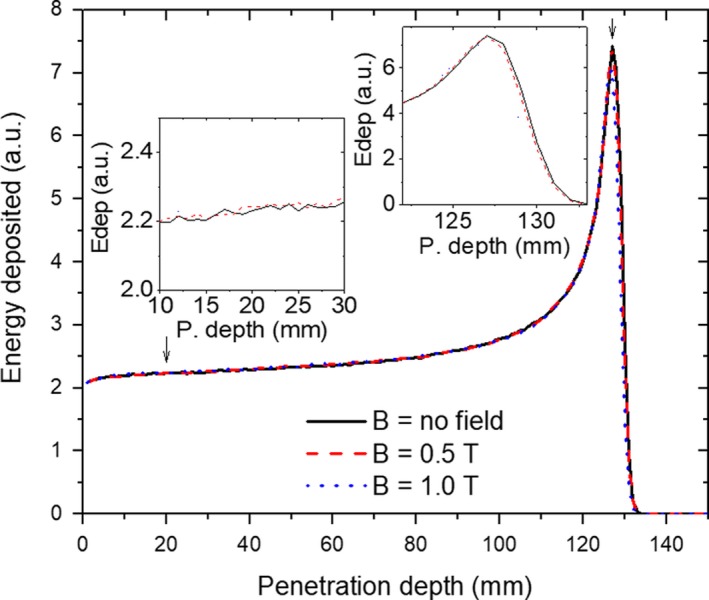
Simulated IDD function for a proton beam of 148.2 MeV passing through a magnetic field region of 0T (solid lines), 0.5T (dashed lines), and 1.0T (dotted lines). The insets provide a zoom of the plateau region where the absolute calibration was performed as well as a zoom of the Bragg peak region. [Color figure can be viewed at http://wileyonlinelibrary.com]

The effect of the magnetic field on dose distributions alongside the beam path in the PMMA phantom for the 148.2 MeV proton beam used for absolute dose calibration was predicted with MC simulations and is shown in Fig. 2. Ranges are almost identical for 148.2 MeV entering the dipole magnet independently of the field strength. Local dose differences in the plateau region caused by the external magnetic field were less than 1% for all analyzed energies and field intensities.

### 3.B Dose response curves

**Figure 3:**
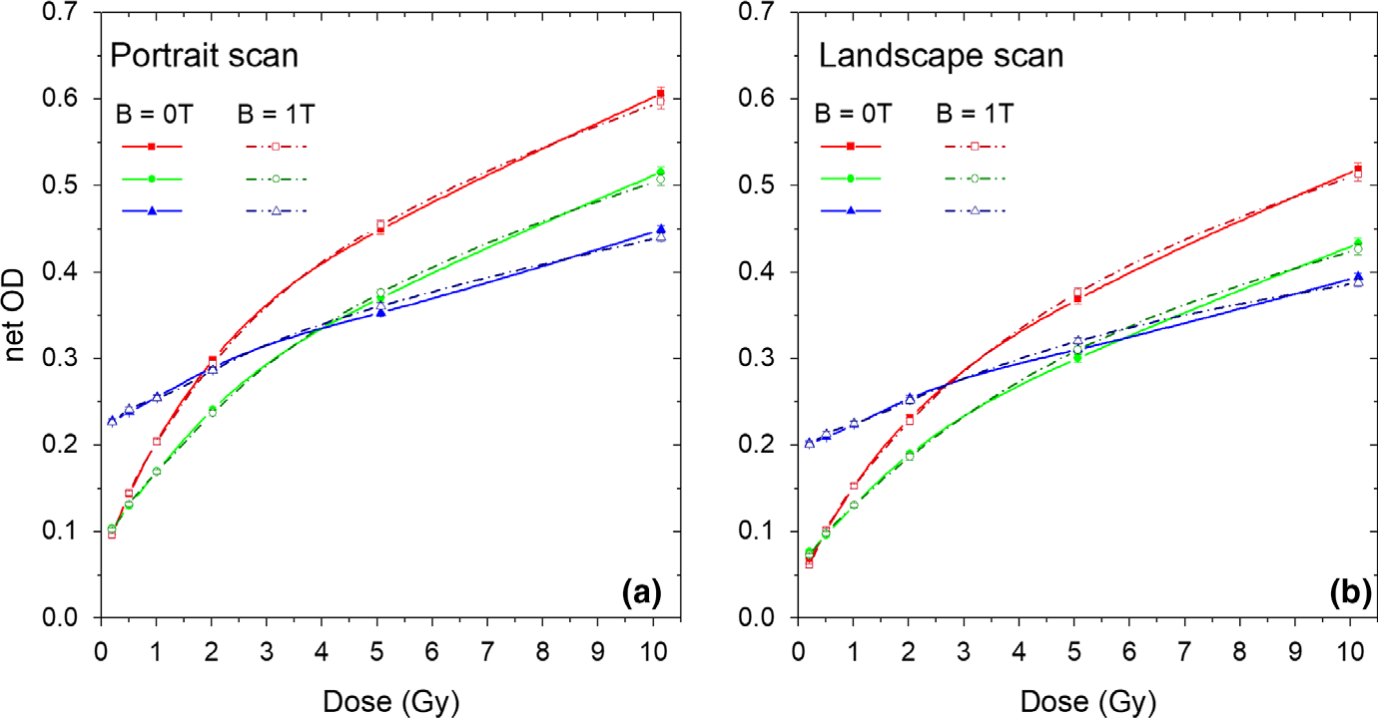
Net optical density for EBT3 films irradiated in the presence (dashed lines) and absence (solid lines) of an external transverse magnetic field scanned in portrait (a) and landscape (b) orientation. Red, green, and blue lines correspond to each of the three RGB channels respectively. [Color figure can be viewed at http://wileyonlinelibrary.com]

No significant deviations (assuming a *P*‐value = 0.05) were observed, neither for the mean pixel value, nor for the net OD, nor the SNR due to the presence of the external magnetic field in the analyzed dose region within the limit of our experimental uncertainties (<2%). The homogeneity of the pixel values in the ROI for every film piece was also very consistent. An overview of the influence of the external magnetic field on the EBT3 film response is displayed in Fig. 3 for all the individual RGB color channels and the two scanning orientations, while 1 summarizes the relative differences of the mean pixel value and SNR between irradiations at B = 0 T and B = 1 T using only the red channel for analysis.

**Figure 4:**
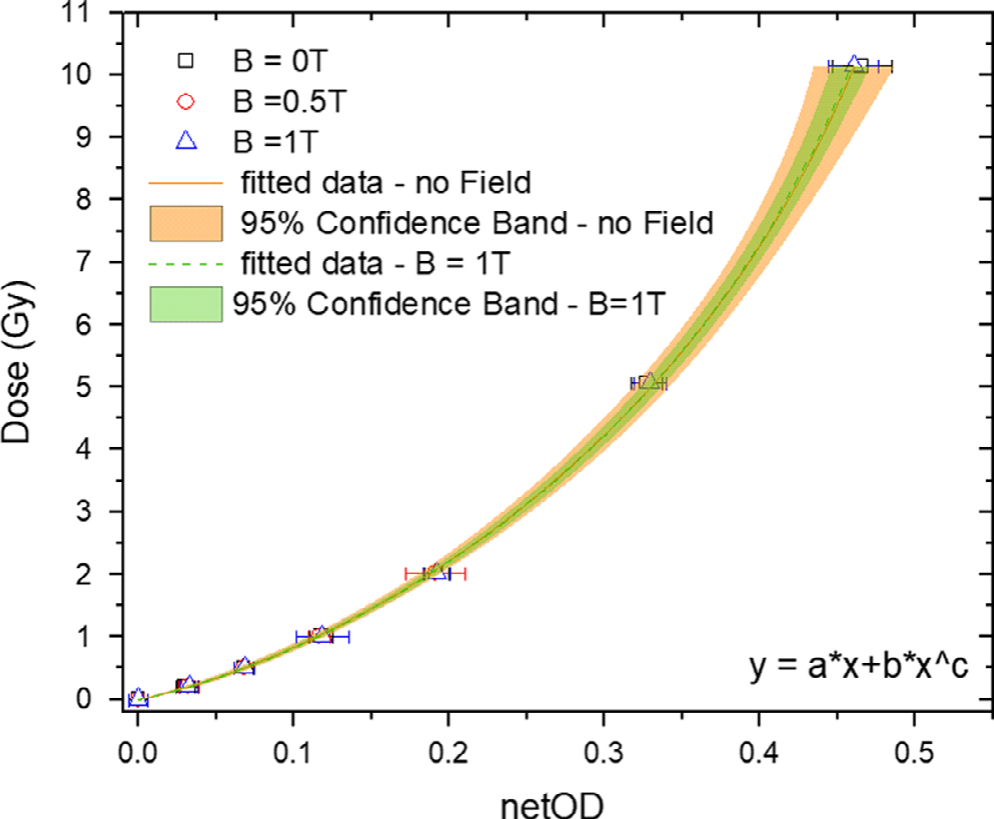
Dose response curves and their corresponding fitting functions obtained for the red channel of the EBT3 films for B = 0T and B = 1 T cases. Confidence bands (95%) are calculated for both fitting functions including the experimental uncertainties. [Color figure can be viewed at http://wileyonlinelibrary.com]

Dose‐response curves are shown in Fig. 4 only for the portrait orientation using the red channel and three different magnetic field strengths. Measurements for the field free case and B = 1 T were fitted by the function described previously in Eq. (2), showing for both cases an R‐square coefficient of unity. The similarity of both calibration functions, obtained for B = 0 T and B = 1 T, was examined using an equivalence test. Relative differences for calculated doses using both fitting functions in a netOD range (0–0.5) were determined. A mean difference of −0.0410% and a standard deviation of 1.6508% was obtained, while deviations between measured and fitted data for all the analyzed configurations were always lower than 1.5%.

### 3.C Dose verification

**Figure 5:**
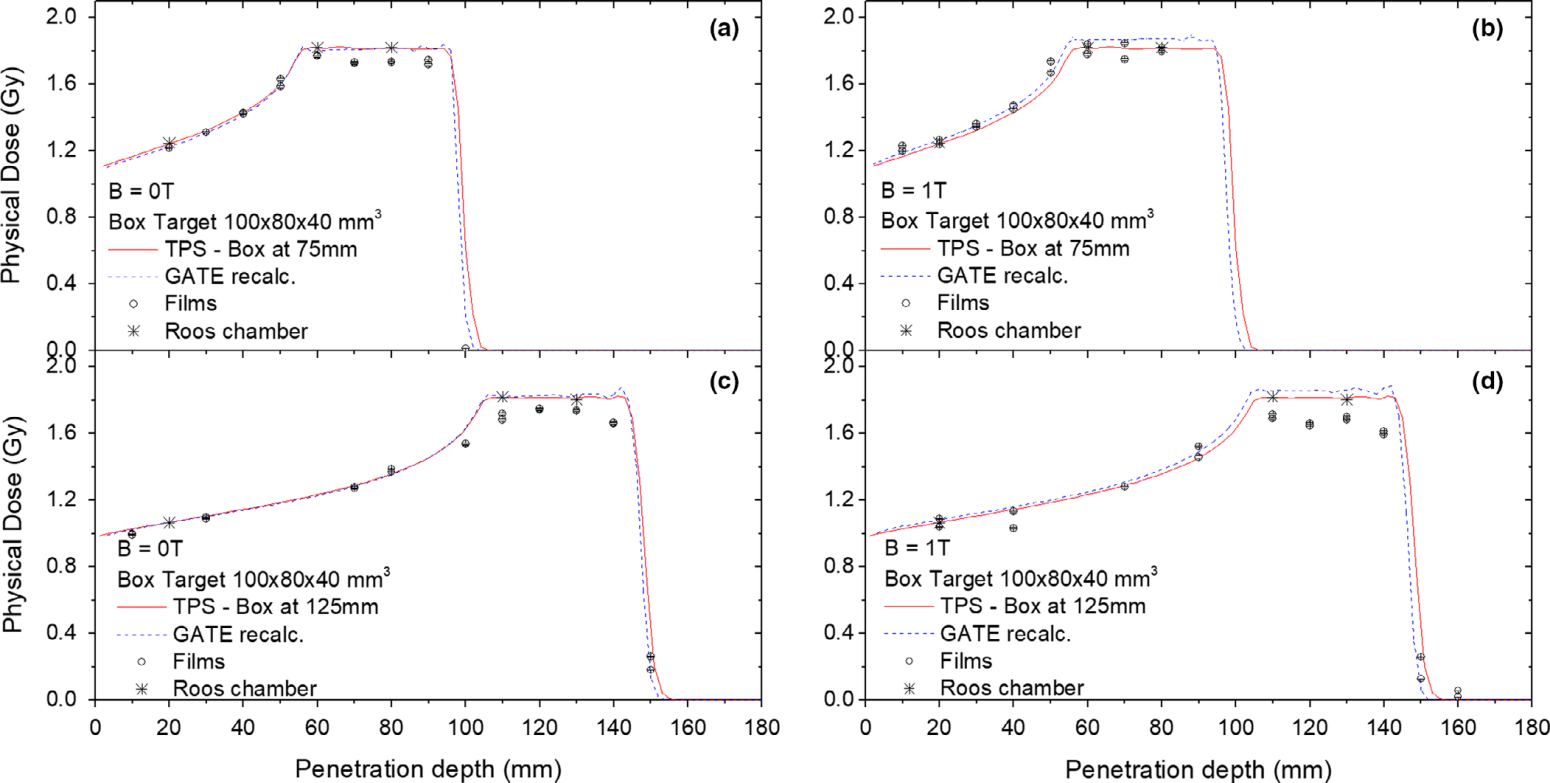
Depth dose profiles for two box targets of 100mm^3^ × 80mm^3^ × 40 mm^3^ centered at 75 mm (top) and 125 mm (bottom) in depth, irradiated with magnetic field strengths of B = 0 T (a), (c) and B = 1 T (b), (d). An underestimation of the measured dose within the SOBP is clearly observed for EBT3 films (black circles) compared to TPS calculated doses (red continue line), Gate simulations (blue dashed lines), and measurements using the Roos ionization chamber (black stars). [Color figure can be viewed at http://wileyonlinelibrary.com]

A comparison between the calculated and measured SOBP corresponding to the two different box target irradiations is shown in Fig. 5. For the target centered at 75 mm depth [Fig. 5(a) and 5(b)], the maximum deviation between planned and measured doses with EBT3 films was 4.5% within the SOBP, while in the plateau region differences were lower than 2%. No noticeable influence of the magnetic field was observed. For the target centered at 125 mm depth [Fig. 5(c) and 5(d)] the largest deviations ranged from −2.3% in the plateau region to −7.3% alongside the SOBP. Discrepancies between the TPS and GATE recalculations, showed a mean deviation of 1.3% (−2.0% to 34.8%) and −1.3% (−5.0% to 57.3%) for non‐field and field cases, respectively, for the target centered at 75 mm depth, while they were −0.2% (−3.5% to 15.4%) and −1.8% (−7.1% to 33.2%) for the target centered at 125 mm depth with the magnet switched off and on, respectively.

A negligible effect of the magnetic field was also found on the absorbed dose determinations with the Roos chamber in the current experimental setup and external fields of B = 0 T and B = 1 T. For the target centered at 75 mm depth the relative differences in the chamber response without/with the field were 0.5%, 0.9%, and 0.8% at 20, 60, 80 mm, respectively, while for the target centered at 125 mm depth, deviations of 0.2%, 1.5%, and 0.9% at 20, 110, and 130 mm depths, respectively, were observed.

## 4 Discussion

The feasibility of EBT3 film for accurate dosimetry of proton beams in magnetic fields was investigated. Considering that no evident field influence was observed on the dose response functions within our experimental uncertainties, film dosimetry offers a very attractive solution for absorbed dose measurement and verification in a future MRPT system. From these results, it can be assumed that guidelines for film preparation, handling, and analysis from proton therapy^3^, ^4^ can also be followed when external magnetic fields are applied.

Previous work for MRXT^13^, ^14^ encountered considerable differences between EBT2 film response for different magnetic field intensities leading to dose differences up to −15% for 20 Gy. More recently, Delfs et al.^12^ using 6MV photon beams showed that for EBT3 film dosimetry, magnetic fields up to 1.42T produced a smaller but still systematic dose under responses of about −2%. From the above‐mentioned studies can be concluded that the structural changes from EBT2 to EBT3 films^29^ improved the detector performance within magnetic field regions for photons. The influence of microstructure changes of EBT2, EBT3, and EBTXD films due to magnetic fields (1.5–3 T) on the response to 6MV photon irradiations was also studied by Volotskova et al.^30^ The observed polymerization and subsequent changes in the net OD were caused by radiation exposure, and nonsignificant changes were observed due to the magnetic field alone influence. For our study using proton beams, no significant modification of the net optical density versus the dose curve at magnetic field strengths was observed within the limits of our experimental uncertainties. A maximal difference between the net optical density with/without the field of 1.1% was obtained for dose levels around 10 Gy. From previous results,^12^ noninfluence of the short film side orientation parallel or orthogonal with the magnetic field lines was demonstrated. For that reason, films were irradiated in the center of the magnet only with their short side parallel to the field lines. The influence of film positioning on the scanner was also negligible, similarly to previously reported.^12^

Absolute dose determination with films in magnetic fields showed under‐response in the Bragg peak region of up to 8% compared with the planned and simulated dose for irradiations with and without the field. This observed quenching effect due to the LET of the protons had been previously described.^1^, ^2^, ^21^, ^23^ Although there are some methods to correct for the dependence of the response of the sensitive layer of the film on the energy‐dependent LET of the particles, within the scope of this work, no correction factor was applied. Independently of the applied magnetic field, EBT3 films showed the same quenching effect in the SOBP toward the distal edge consistently with previously reported data for irradiations without external magnetic fields.^1^, ^2^, ^21^, ^23^ No additional effect was observed in our measurements due to the magnetic field.

Larger deviations between MC simulations and planned doses when the magnetic field is applied [Fig. 5(b) and 5(d)] were observed for the target centered at 125 mm. Treatment plans were generated with the commercial Raysearch 5.99 TPS using the beam model data acquired without external magnetic fields, not accounting for the fact that bent trajectories of the proton beams in the presence of magnetic fields induce a slight retraction of the Bragg peak position and nonsymmetric lateral dose distributions.^18^, ^19^ In this work, two SOBP were analyzed, covering penetration depths from 55 to 95 mm and 105–145 mm in PMMA. The SOBP centered at 75 and 125 mm were created using 52 and 32 energy layers, ranging from 92.8 to 128.4 and 132.6–161.6 MeV, respectively. For these two‐energy range, lateral beam deflections between 6.3–11.3 and 12–17.3 mm as well as Bragg peak shifting in the ranges of 0.5–0.6 and 0.7–0.8 mm were predicted with MC simulations. As a consequence, dose deviations alongside the SOBP were expected between the TPS and GATE recalculations. Bragg peak retraction and lateral shifting of proton beams due to external magnetic fields were theoretically investigated previously^18^, ^31^ and validated by measurements.^32^ As previously demonstrated, the higher the required energies of the individual proton pencil beams to cover the target, the higher the lateral beam deflection and Bragg peak position longitudinal shifting. Therefore, more pronounced differences between established TPS and MC simulations are anticipated for targets located at deeper depths. As previously discussed, to assure accurate treatment planning of protons in magnetic field regions, the currently used dose calculation algorithms need to be adequately modified.^19^, ^31^

Magnetic field maps used through this work were generated from measured data by the manufacturer in a volume of X, Y, Z = 700, 420, 700 mm^3^, assuring that the complete fringe fields were accounted for. The influence of the scanning magnets from the PBS delivery system was not accounted for, considering that they are located 7000 mm away from the room isocenter. More realistic evaluations of the resulting magnetic fields from the interaction of the beam line magnets and the research magnet used through our experiments could be obtained using a finite element model.^33^ However, magnetic field intensity and homogeneity were measured at different reference points inside and outside the magnet, showing a close to perfect agreement with the data reported by the manufacturer.

## 5 Conclusions

The feasibility of accurate EBT3 film dosimetry for a possible future MRPT system was analyzed. The mean pixel values and net optical densities of the films were evaluated for proton beams in the presence of different transverse magnetic fields, showing negligible influence of the field strength on the dose response functions in the proposed experimental setup, within our measurement uncertainties. From these results, EBT3 films show very attractive properties for proton beam dosimetry within magnetic field regions. Although films have higher uncertainties in dose determination than ionization chambers, the absence of a significant influence of the magnetic field on their response represents a potential advantage over other detectors. However, similarly as in the absence of a magnetic field, quenching corrections for protons are required for depth dose measurements of pristine and spread‐out Bragg peaks.

The proposed method in this work offers a viable solution for dose measurements of proton beams within magnetic fields up to 1T, assuring negligible influence on the film response. The calibration method and experimental setup used throughout all our experiments is advised in order to guarantee an efficient and reproducible use of films.

## Conflict of interest

The authors have no conflict of interest to report.

6

**Table 1:** Relative differences (%) from the mean pixel value and the SNR for EBT3 films irradiated with B = 0 T and B = 1 T scanned in different orientation. Deviations are calculated using the nonfield irradiation as reference values.

Dose (Gy)	Landscape orientation		Portrait orientation	
	ΔMean‐PV (%)	ΔSNR (%)	ΔMean PV (%)	ΔSNR (%)
0.2	1.3	2.3	0.8	2.9
0.5	−0.3	−1.9	−0.4	3.2
1.0	<0.1	−3.5	0.2	0.8
2.0	0.8	<0.1	1.0	2.7
5.0	−1.7	1.9	−1.2	−1.6
10.0	1.4	−1.7	2.1	2.5
